# Being a member of a novel transitional case management team for patients with unstable housing: an ethnographic study

**DOI:** 10.1186/s12913-022-07590-6

**Published:** 2022-02-19

**Authors:** Dailys Garcia-Jorda, Gabriel E. Fabreau, Queenie Kwan Wing Li, Alicia Polachek, Katrina Milaney, Patrick McLane, Kerry A. McBrien

**Affiliations:** 1 Department of Family Medicine, Cumming School of Medicine, University of Calgary, Calgary, Canada; 2 Department of Community Health Sciences, Cumming School of Medicine, University of Calgary, Calgary, Canada; 3 Department of Medicine, Cumming School of Medicine, University of Calgary, Calgary, Canada; 4 Emergency Strategic Clinical NetworkTM, Alberta Health Services, Edmonton, Canada

**Keywords:** Marginalized or Vulnerable populations, Homelessness, Case management, Patient care team, Interprofessional relations, Frontline providers, Case management workers, Qualitative study, Canada

## Abstract

**Background:**

Homeless and unstably housed individuals face barriers in accessing healthcare despite experiencing greater health needs than the general population. Case management programs are effectively used to provide care for this population. However, little is known about the experiences of providers, their needs, and the ways they can be supported in their roles. Connect 2 Care (C2C) is a mobile outreach team that provides transitional case management for vulnerable individuals in a major Canadian city. Using an ethnographic approach, we aimed to describe the experiences of C2C team members and explore their perceptions and challenges.

**Methods:**

We conducted participant observations and semi-structured interviews with C2C team members. Data analysis consisted of inductive thematic analysis to identify themes that were iteratively discussed.

**Results:**

From 36 h of field observations with eight team members and 15 semi-structured interviews with 12 team members, we identified five overarching themes: 1) Hiring the right people & onboarding: becoming part of C2C; 2) Working as a team member: from experience to expertise; 3) Proud but unsupported: adding value but undervalued; 4) Team-initiated coping: satisfaction in the face of emotional strain, and; 5) Likes and dislikes: committed to challenges.

**Conclusions:**

A cohesive team of providers with suitable personal and professional characteristics is essential to care for this complex population. Emotional support and inclusion of frontline workers in operational decisions are important considerations for optimal care and program sustainability.

**Supplementary Information:**

The online version contains supplementary material available at 10.1186/s12913-022-07590-6.

## Background

Homeless and unstably housed individuals are systematically underserved by health and social care [1, 2]. In addition to experiencing greater and more complex health needs than the general population, these individuals face barriers in accessing health care [3–5]. Even when accessible, primary care and social services often do not meet the needs of vulnerably housed individuals in the community [4, 6, 7]. As a result, these individuals turn to acute hospital care at a higher rate [8, 9], remain hospitalized longer [10], and experience higher age/sex standardized mortality and a shorter life expectancy [11].

Case management programs are widely studied for their effectiveness in complex care [12]. These services have been shown to improve outcomes for homeless and unstably housed patients with complex needs by coordinating care across healthcare settings and over time [13–15]. In addition, community-based programs that include patient navigators can improve access and lead to more appropriate health care utilization [16–19]. Patient (health) navigators are patient-care facilitators that act as mediators between patients, healthcare, and social and community services [20]. However, while rates of burnout, post-traumatic stress, and vicarious trauma among frontline providers in the homeless sector are extensively reported [21–23] little is known about the experiences of providers, their needs, and factors that can support them in their roles.

Connect 2 Care, known as C2C, is a mobile outreach program that provides transitional case management for homeless and vulnerably housed individuals. The program combines elements of intensive case management with health navigation, and aims to improve the quality, access, and coordination of care for complex patients [24]. Understanding C2C team members’ experiences may explain how daily activities, services, and supports provided to clients translate into care outcomes and identify ways of developing successful programs. In this qualitative study, we aimed to 1) describe the experiences of C2C managers and frontline providers (registered nurses and health navigators) and 2) explore how the emotions, perceptions, and challenges of frontline providers shape their practice. Focusing on the implementation of a program from the perspective of the team has the potential to highlight how provider characteristics, agency, and activities influence program outcomes.

## Methods

### Study setting and design

C2C is a mobile outreach team developed in partnership between a community health centre that provides health, education, and housing programs and a housing agency that provides shelter, medically assisted withdrawal management facilities, and housing services to individuals in Calgary, Canada. C2C provides intensive case management, transitional care, advocacy, patient navigation, and care coordination to patients with unstable housing and high acute care use (three or more emergency department or urgent care presentations, or two hospitalizations within a year). Most C2C clients (approximately 80%) were homeless (living on the street, in shelters, or in temporary accommodations), and the remaining minority was housed. The C2C frontline team is comprised of registered nurses (RNs) and health navigators (HNs) with collective expertise in mental health, chronic disease management, and addiction, as well as extensive knowledge about local social programs including community health, housing, income support, and legal resources. One C2C nurse offers palliative care and support for clients at end of life. Depending on specific circumstances, C2C staff work closely with clients, care givers and the heath care team on hospital discharge planning, connection with housing supports, primary care and substance use treatment, and following up to assist with service navigation and access [24].

We conducted a mixed-methods evaluation of C2C beginning in September 2016, in parallel with program implementation and refinement. Preliminary results were leveraged to inform program sustainability and expansion*.* This article reports an analysis of qualitative data with a focus on team member experiences. The University of Calgary Research Ethics Board approved this study, and all participants provided written informed consent.

We adopted an ethnographic approach to understand the experiences of C2C team members. Ethnography is the study of culture, “social interactions, behaviours, and perceptions that occur within groups, teams, organizations, and communities.” Its overall aim “is to provide rich, holistic insights into people’s views and actions […] through the collection of detailed observations and interviews” [25], to understand the object of study. Our fieldwork focused on organizational elements and how team members worked to care for clients.

### Sampling strategy

All C2C team members, including managers and frontline providers (RNs and HNs), were eligible for inclusion. We invited participants via email to participate in field observations and/or in-person semi-structured interviews. The researchers who participated in the data collection were unknown to study participants.

### Data collection

We conducted participant observations to better understand the daily activities and interactions of C2C team members. Observations were conducted from February to June 2018 at varying days and times during C2C service hours (Monday to Friday, 8:00 am to 4:00 pm). The trained observer spent time as a passive observer shadowing one team member per session of three hours each. Since the observations focused on C2C frontline providers and their activities, we informed clients beforehand about the presence of the observer but did not include any client-related information in analyses. As such, clients were not asked for informed consent, but were given the option to decline having the observer present during their encounters. Immediately after observations, the observer translated brief notes taken in the field into comprehensive notes that were used for analysis.

We also conducted semi-structured interviews using an interview guide (Additional file 1) with C2C team members during two distinct periods—August to October 2017 and September to October 2018—to gather data about organizational culture, how team members experienced their roles, and how the growth of the program over time impacted them. Interviews at two time points were also used to capture differences between those who were involved in the program’s early stages and those who subsequently joined the team. Interviews were conducted at times and locations convenient to participants and lasted between 25 and 50 min. The interview guide included broad, open-ended questions about the participant’s role within the program, their experiences, likes and dislikes about the program, and early successes. Probes were used if needed to clarify questions and elicit additional details. To describe study participants, we collected sociodemographic data including year of birth, gender, and education from all participants. All interviews were in-person and audio recorded. A research assistant transcribed interviews verbatim and removed all identifying information. All transcripts were reviewed by the interviewer or another research team member, and each participant was identified using a code to ensure confidentiality.

### Data Analysis

The first author conducted inductive thematic analysis of all observation field notes and five interview transcripts to develop a preliminary codebook that consisted of code names, their definitions, inclusion and exclusion criteria, and examples of text from the transcripts [26]. The preliminary codebook was discussed with members of the research team over several meetings where codes and their definitions were scrutinized, clarified, and enhanced accordingly. Using this codebook, three additional researchers participated in coding so that all interview transcripts and observation field notes were coded independently by two researchers. During this phase, the coding was mostly deductive but inductive coding was considered if new codes were identified. We inductively developed themes that were iteratively reviewed and scrutinized by the research team.

We determined Interrater Reliability (IRR) using the Cohen Kappa coefficient to guide the discussion and review of the coding process. Since there is no single threshold for an acceptable value of kappa [27, 28], we reflected on codes with a Cohen’s K score lower than 0.61, which has been considered a substantial agreement [29]. Other codes (with higher Cohen’s K) were also discussed if deemed necessary by any of the researchers. We resolved coding differences via discussion until agreement was reached. Transcripts were recoded if the coding, definitions, or descriptions changed. We used NVivo 12 for Mac (QSR International Pty Ltd., Victoria, Australia) for coding, theme development, and calculating the IRR. We followed the standards for reporting qualitative research (SRQR) [30].

Representative quotes from participants using pseudonyms were included to support analytical findings. We used data from all participants for theme abstraction; however, excerpts from participants in management positions were not used in manuscripts to protect their identity. We presented the interpretation of our findings in two C2C operational meetings, where we received feedback from team members to support the validity of the analyses.

## Results

All frontline team members hired during the times of data collection participated either in observations, interviews, or both. The number of providers was not the same at all data collection times due to staff turnover and stages of implementation of the program. In total, 36 h of observational data were collected from 12 3-h observation sessions with eight C2C team members. Observation locations included the C2C program office, clients’ housing, hospitals, and pharmacies among others. A total of 15 interviews (six during the first period of interviews and nine during the second) were conducted with 11 C2C team members (two managers and nine frontline providers). Both managers and two frontline members were interviewed twice. Six team members, including those who were interviewed twice, had worked for the program longer than two years at their first interview or first time observed. Two team members worked for C2C between one and two years, two for between six and twelve months, and two for fewer than six months. Table 1 shows participant characteristics.

**Table 1:** Participant Characteristics (*N* = 12)

	*N*
Gender (self-identified)	
*Male*	7
*Female*	5
Age, Range	30 – 57 (Mean 38.5)
Education	
*Technical certificate*	1
*Bachelor’s degree*	8
*Master’s degree*	3

**Fig. 1:**
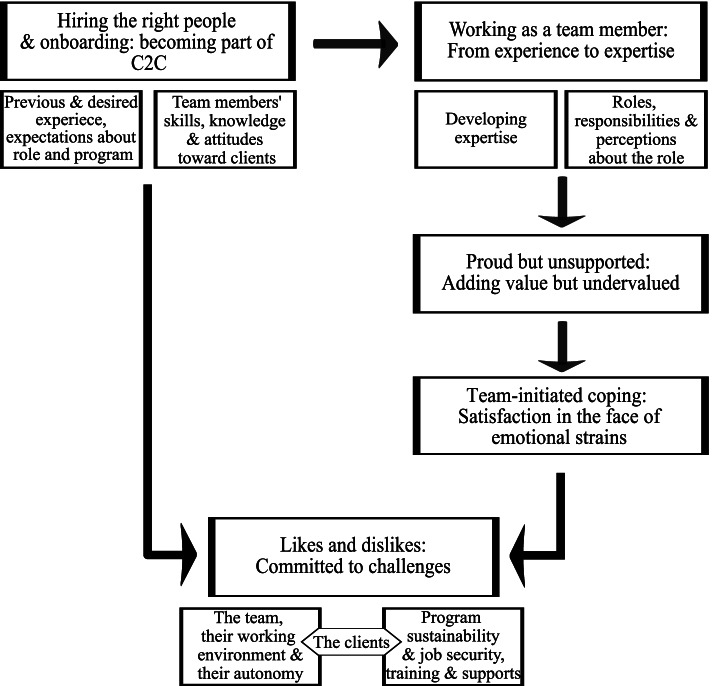
The Experience of Being a C2C Team Member. Overview of Themes and their Relationship

We identified five main themes relating to C2C team members’ experiences and perceptions: 1) Hiring the right people & onboarding: Becoming part of C2C; 2) Working as a team member: From experience to expertise; 3) Proud but unsupported: Adding value but feeling undervalued; 4) Team-initiated coping: Satisfaction in the face of emotional strain; and, 5) Likes and dislikes: Committed to challenges. Fig. 1 presents a thematic overview. In general, most C2C team members described strong feelings and self-awareness about their value in the program, the population they serve, and the wider community. Team members expressed being simultaneously committed to their clients, yet hopeless and frustrated when they felt unsupported by management or social systems in meeting clients’ needs. This ambivalence represents a team that is proud of their work but challenged by the nature of the work.

### Hiring the right people & onboarding: becoming part of C2C

When making hiring decisions, *C2C* managers specifically looked for candidates with previous experience as an outreach nurse or shelter worker. All frontline providers were hired by *C2C* but reported to one of the two partner agencies depending on their role; RNs were employees of the community health centre while HNs were employees of the housing agency. Previous experience was both essential and a motivation for the job; team members were able to both identify their clients’ needs and recognize gaps between the acute care system and community services (e.g., primary health care and social services), working to bridge the two to address clients’ concerns. A health navigator described:

*I have worked at [housing agency] for four years in another form of outreach […] moving to C2C, was a natural progression from being more crisis-intervention outreach to more focused outreach that dwells with the health concerns our clients face. (Alex, HN)*

Despite their experience, both C2C managers and frontline workers noted a steep learning curve once in the C2C role. To adapt to their new role, most team members drew primarily on their education or previous work experience rather than job-specific training. Team members described having to be self-sufficient in navigating the logistics of C2C and, notably, some felt less confident after more job exposure as they encountered situations they had not expected. In addition, interviewees noted disparities in previous experience between team members. Jordan, an RN, expressed:

*If I didn't have that background, it would have been much harder, and I know speaking with some of the other health navigators who have less varied experience, I felt like there was maybe, they weren't quite as prepared. And I think that showed, like it showed in the work that they were able to get done.*

Frontline providers stated that flawed onboarding was partly responsible for their incomplete preparedness for the job. HNs who started earlier in the program received more intensive training than more recently hired staff who were trained primarily through shadowing team members in the same roles. Some frontline providers felt that they “were thrown into” the role without a strong onboarding or training process. As Jordan also explained:

*It’s really important to have that, that proper onboard and training right at the beginning. […] I think for a couple of the, the health navigators when they started […] they would follow one person around for three days and witness, you know, a small percentage of the many things that we do and then say like okay go. […] I think it’s been overwhelming for some people for sure.*

The team members’ main concern around onboarding was insufficiency of training and the potential negative impacts this had on client care. All frontline workers expressed the need for more HN training specifically. For example, HNs emphasized that it would have been helpful to have training in motivational interviewing techniques and trauma-informed care. Team members offered other suggestions about “good to have” operational elements that would improve their performance, including having business cards, a corporate email account, and earlier access to the electronic medical record*.*

*I think we do a disservice to clients if we don’t prepare and train staff appropriately from the beginning because that's someone's life that you're working with and just because you don’t know what you're doing, they shouldn't be, you know, worse off because you just didn’t know that one thing that we could have easily told you. (Sam, HN)*

*I had motivational interviewing from before […] and then there was one Mental Health First Aid so I took that but that was it. But they were not offered to me as part of the job training. […] Trauma-informed care would be one that I feel would be a very important part to this job. (Alex, HN)*

*C2C frontline meeting: The team discusses different training sessions and one of the HN says “in-service was the best training that we have ever done and should do it again as the new HNs that don’t have a lot of experience would benefit from it” (Observation field notes)*

Despite a perceived lack of adequate onboarding, a comprehensive set of inherent skills, attitudes, and knowledge was evident in observations and team member interviews. For example, one provider shared that it was essential for frontline members to be able to build rapport and communicate effectively with their particular clients, embrace clients’ realities, and understand the barriers to care they face as a personal quality team members needed to possess. One of the RNs explained:

*You couldn’t put just anybody into these roles and expect the same results. Um, because it needs to be, there needs to be a certain type of person that a) can take on the workload, because it’s not easy, b) take on the clients and, and the traumas and stuff that they experience and express on a day-to-day basis, and [laughs] you know, not carry, not take that on themselves, that’s why the program works. (Logan, RN)*

In observations, frontline providers showed sensitivity, compassion, and empathy with their clients’ needs and problems. C2C staff used various social techniques to approach and connect with clients. Their familiarity with the population was manifest through body language and use of popular vocabulary, demonstrating cultural competency and respect for different social backgrounds. Several instances were reflected in the observation field notes:

*The HN [identified as Lee] had to go through several rooms upstairs [at a community shelter] to find where his client was. He found him in the living area upstairs sitting at a desk by himself. The client smiled when he saw the HN and said, “I was hoping to see you”. The HN then smiled back and got on one knee to talk to the client about how he was unable to pay his rent last month.*

*The RN [identified as Casey] arrives at a patient’s room in a hospital. The client looks confused and tells the RN that she doesn’t remember meeting [her]. Casey explains to the client that the last time they met, she [client] was pretty sick and it's reasonable that she wouldn’t remember. […] After conversing about the patient condition, stay at the hospital, and medications, the client mentioned: “I need a pain reliever, or I can’t function with my neck and hip pain. […] without the pain relief I’m very crabby”. The RN nodded and said: “Of course you are crabby, you are in pain. I would be crabby too”.*

### Working as a team member: from experience to expertise

Frontline members described their roles and responsibilities as broad; as one RN (Drew) mentioned, they are *“a jack of all trades”.* Roles were noted to be flexible and depended on clients’ needs, with daily tasks including locating clients, advocating for and coordinating care at healthcare or social agencies, accompanying clients to appointments, and hospital discharge planning. Program evolution over time impacted frontline members’ expectations and perceptions about their roles. For example, frontline staff hired early after program launch noted an unexpected emphasis on meeting clients’ housing needs rather than focusing on their medical and health concerns. Those who were interviewed twice noted in the later interview how addressing their clients’ housing and social needs first was essential to managing their health concerns. In contrast, when recently hired frontline providers were asked to reflect on their expected and perceived roles, most found the actual role matched their expectations.

*I thought what we were going to be doing first, was acting like a case manager for peoples’ medical needs and only focusing on that, but what it’s turned into is much different, we end up working a lot on the social issues at least as health navigators, so looking at connecting with housing programs, working on funding and income support stuff, so we’re doing a lot more of the hospital social work sort of role. (Lee, HN, 1*^*st*^* period of interviews)*

*I mean at the beginning [pause] we thought, I know myself specifically, that we would be dealing with a bit more the medical stuff […], then it’s a lot more about case management around housing and all the sort of social determinants of health more so than the actual health. (Lee, HN, 2*^*nd*^* period of interviews)*

*I was surprised at the amount of housing work that we did, although I guess not that surprised 'cause of the social determinants of health, you have to do that to make people be healthier. (Sam, HN, 2*^*nd*^* period of interviews)*

Team members described feeling proud about the uniqueness of the role that they actively developed and defined. However, they stated that their daily activities could have been better supported through guidance from a senior team member, or perhaps a resource guide with solution pathways for common client issues. Frontline staff expressed a shared sense of satisfaction about their increasing confidence and capacity over time, as they intentionally increased their knowledge and skills to better serve their clients. It is evident that team members’ expertise developed over time as they overcame various challenges experienced on the job.

*I learned, and had to learn, quickly which I did because it was just two of us and we had a huge case load so yeah. […] But prepared, I like to think I am but there's definitely something that throws me every day, yeah. Which I love, I like, this is why I love the job, I like challenges, I like thinking on my feet and figuring out what to do and yeah, I guess whatever can help the client I'll bend over backwards to do so, yeah. (Casey, RN)*

*I have learned […] real and substantial ways of actually helping [the clients]. (Alex, HN)*

Working as a team, C2C frontline providers created a cohesive and safe culture whereby they could share knowledge and opinions, working collaboratively to meet clients’ needs. An integrated team was considered essential as individual roles were refined while facing daily frontline challenges. Observed teamwork was influenced by frontline providers’ individual backgrounds and previous experiences as they intentionally involved each other depending on specific expertise. This was confirmed in interviews, Alex (HN) explained:

*Because everything is in development, sometimes when you are not sure what you need to do, you don’t really have […] anyone that you can really go to, someone in a more of a senior role to ask […] I kinda see maybe that’s another reason why the frontline team is so tight, works so well together is because, I don’t know how to put it [long pause] I don’t know if guidance is the right word, lack of guidance.*

Interprofessional collaboration between HNs and RNs was evident and devoid of hierarchical conflict. Providers regularly discussed, advised, and supported each other’s cases to support their clients’ care. They also delegated tasks amongst the entire team and routinely filled in for members who had competing client commitments or were otherwise unavailable.

*We all have a different knowledge base that we can sit down and actually come up with a good solution. We can disagree, we can disagree for hours and hours, but at the end we always come up with ways to deal with the challenges we face. (Casey, RN)*

*What I liked most was the close-knit teamwork. […] [Team members] had very good communication, we were always very open on what’s going on with our clients. I really liked that kind of shared responsibility. (Jules, HN)*

*I think that’s something we do well, is meeting weekly and sort of just helping each other work through our challenges and the caseload. (Lee, HN)*

*C2C frontline office: Jordan (RN) asks Lee (HN) about a client, while pulling up the client’s documents on wolf [electronic medical record]. After discussing the client’s situation, the HN tells the RN that it might be good to call the MAID [Medical Assistance in Dying] navigator for this client and passes on the MAID navigator’s number. (Observation fieldnotes)*

### Proud but unsupported: adding value but undervalued

Team members reported valuing the program and work, and recognized their contributions to clients, the broader community, and the healthcare system. Frontline staff described seeing themselves as experienced holders of valuable knowledge accumulated over time. All members agreed that the C2C program positively impacted clients, the healthcare system, and other community agencies, thus providing both meaning and satisfaction.

*I think we do fantastic work and even the numbers show like what we've done. I think we've made a huge difference and I hope it continues. […] Not only for clients. Yeah. I definitely think […] a lot of hospitals and community agencies rely on us. (Casey, RN)*

*I think it’s easy to see individually how we make a difference 'cause our clients you know we can see them moving forward and we can see progress happen, and then I think it was also nice to sort of see systemically when we got the research data back that we were making an impact […] to see that all those kinds of little things that we do with our clients actually do make a big impact which is kind of nice to see.* (Sam, HN)

To some extent, however, frontline providers felt unsupported, powerless, and disconnected from their managing organizations, as well as excluded from decision making. These sentiments may have developed over time as they were not evident during the initial interviews. As Lee, an HN expressed in his second interview: *“I think we’ve definitely lost a bit of our voice lately”.*

Although frontline C2C members relied on their cohesion to work effectively, they expressed the need for clarification of program goals with upper levels of the program. Frontline staff also shared that acknowledgement of their contributions would ultimately improve client care. Some frontline members described that discussing solutions for service issues were unsuccessful because they felt unheard, or outright ignored, by the managing agencies. Strained communication and lack of feedback from management made frontline members feel their concerns and suggestions were invalid.

*I think as the frontline team we're all on the same page as the nurses and the health navigators, but I think when it gets above us then people have different expectations of what they think our program should be so, maybe having more of a voice in that would be kind of nice. (Lee, HN)*

*I think we’ve forgotten a little bit about who’s at the center, and that’s the client, and as a result of that, we’ve forgotten to support the people that are helping those clients. […] I think supporting the frontline staff a whole lot better, really making sure that they feel that any concerns that they have are validated, that they’re valued, that they have the resources that they need. As far as training goes, to as far as, you know, a really very clear line of communication of who to go to when you have a concern. And then a feedback loop to tell you that that concern has been dealt with, in a good way. That’s I think what’s missing and I think that’s where people are left a bit floundering and in the dark and feeling a bit undervalued and frustrated. (Logan, RN)*

Managers, for their part, described a desire to provide support and be connected with the team, by providing guidance and being present at meetings, but also allowing the team a certain level of autonomy that recognized their expertise. Managers also noted some challenges with C2C being a new program, and one under study, with operational and decision making processes still under development.

### Team-initiated coping: satisfaction in the face of emotional strain

Two C2C team members reported feeling burned out and others described instances where they felt stressed, overwhelmed, or exhausted. One of the main factors identified in relation to these feelings was the burden of not meeting their clients’ needs due to staff or resource limitations within and beyond C2C. Team members also reacted to specific challenges (e.g., lack of compliance or follow through) they faced with their clients.

*I'm gonna be honest, a lot of us come into work and […] say I just can't do this anymore, like this is so hard, like I just need to go and work in a hospital where I know somebody's coming to take over my shift. (Casey, RN)*

*I think it's hard to not get discouraged sometimes when clients don't follow through and you set up all sorts of things for them and then it falls apart. (Drew, RN)*

Despite these feelings, frontline providers also expressed instances where they felt content and enjoyed their job. They were both intrinsically and extrinsically motivated to continue their roles, finding personal satisfaction connecting with underserved populations, as well as witnessing positive changes in their clients’ lives.

*When I go and visit my clients in a house that I’ve housed them in, like even just showing me where they cook a meal in the afternoon is like a huge thing to them and I think it’s, like it’s heartwarming. (Casey, RN)*

*I think it’s pretty amazing to kind of be able to support our clients through some pretty difficult stuff […] I feel good that we’ve you know had some success in like getting people to get treatments and to take better care of their [health] […] it’s been nice to you know see some people get housed and see some people do really well that I’ve known for a long time here just on the mat [at the shelter]. I feel pretty good about it. (Sam, HN)*

C2C’s collaborative teamwork was a core element spoken about in all interviews and evident during observations. Frontline members described a working environment that was strengthened by comradery. Themes of solidarity, trust, and developing bonds beyond typical working relationships were consistent among all participants.

*I literally love getting up for work and coming, like if I didn't work with the team that I work with, I don't think I would like my job as much as I do. (Casey, RN)*

*I think on the whole like working as a team you get that sense of comradery, you get the different perspectives, you also have like you know, back up. […] For example, I went to a home visit and I thought the guy was for sure dead, so I was like I'm going to knock on the door, and I would love it if someone else is there. […] You say things like that, when you are part of a team, someone’s like yeah okay let's do that. So I think the team-based approach is for sure the way to do it. (Jordan, RN)*

*I really like the comradery, the teamwork [pause]. Everybody gets along. (Jaime, HN)*

The environment built by the frontline team was considered a crucial element in coping with the emotionally demanding job. Aside from the latent support of these relationships, most frontline providers shared that their caseloads and client responsibilities left inadequate opportunity to properly work through traumatic events. Participants believed routine debriefing should be integrated into the program structure to support team members’ own health. Frontline providers requested dedicated time to engage in team building activities, recognize team contributions, and for general encouragement and support.

*Our team is really great so if you have a really bad day you can talk to anybody, they'll take some time and hang out with you. […] often if one of us has a really bad day we just sit in our outreach office at the end of the day and talk about it, we've been here for an hour or two after work sometimes just hashing things out and talking about someone’s day to sort of try and mitigate that […] I feel supported on my team. (Sam, HN)*

*Having designated team-building time and debrief time, I think that would be really helpful and I think probably it would be helpful to have someone, take us a bit through a more of a structured, more of a structured debrief. (Jordan, RN)*

*[…] trying to support one another and talk about the successes we've had of the week, not just the ones that haven't worked out. We try to focus on that and some weeks that's easier than others for sure. (Drew, RN)*

C2C managers agreed frontline members required increased emotional support given the challenging nature of their work. Managers mentioned that support was available through the partner agencies; however, the frontline staff expressed doubts that people outside their specific roles could adequately understand their experiences and losses. Lee (HN) explained: *“As a frontline team we support each other. I mean we definitely can do a better job with debriefing and having set times to sort of chat about that thing, but I mean we do our best in this role”.* One frontline member mentioned that a guided debrief by a professional external to the team and the partner agencies could be helpful.

### Likes and dislikes: committed to challenges

Program limitations were noted in that C2C operates only during weekday business hours and is a relatively small team providing care to a high need, complex population. One of the RNs mentioned:

*We kind of get to a capacity where we can't take on any more people because we're already busy managing these many individuals that are already quite complex. I think the team's capacity is something that might limit our success down the road […] But I would say [pause] yeah like the manpower of the team, the human resources component and then the housing are the two biggest things that will impact our ability. (Drew, RN)*

In addition to capacity challenges, C2C’s operational dependency on grant funding was a source of insecurity for some team members. Frontline staff expressed worries about the program’s sustainability and their own job security. Jaime (HN) expressed: *“Being that it’s a grant, it’s a little bit nerve wracking that a year from now I may or may not have a job.”*

Team members were motivated by the autonomy of working with C2C, including the flexibility of practice they were able to maintain with their clients. Frontline staff were able to set priorities and organize their workday based on their clients’ needs, not on pre-established tasks or other restrictive structures.

*I definitely felt like I had the freedom to do what I thought was the best (Jules, HN).*

*I like that it’s [pause] fairly independent. I mean there’s accountability but it’s not like, it’s for us to decide our day to day, prioritize. So, I like the, I guess the autonomy. A little bit more autonomy than most other positions, where it’s like oh, you do this, this, this, this, at this time, or you have a list of tasks. (Jaime, HN)*

The clients themselves were one of the team’s main motivators. Participants said they liked their clients and loved helping underserved populations. However, client complexity was also a consistent challenge. Connecting and establishing bonds with clients, keeping them engaged and motivated, and considering their personal interests were obstacles described by participants.

*I have wanted to work with this population my whole life. I have always taken an interest […] I do this work because I love this population and these are my folks. (Jules, HN)*

*They [clients] lose their cellphones, their cellphones are stolen, they don’t have money, they can’t pay for their cellphones, maybe they never had one in the first place. And they’re living in shelters, so of course, that is what makes those health navigators wizards, because they find people without any of that information […]. But even then, it is challenging. You just lose people, you have people living in cars, and no way to get a hold of them. (Logan, RN)*

## Discussion

In our ethnographic study of a novel community outreach program, we learned that it was essential to hire the right people for the job, but that training and adaptation were still required; that teamwork and a culture of mutual respect and safety enabled the team to effectively execute their roles; and that working within the constraints of the health and social system presented challenges that are difficult to overcome. C2C team members also shared their early successes, personal struggles in the program, and professional growth as they defined their roles. Team member experiences have enriched our understanding of how a program like C2C operates and the critical role that team members have in shaping the services delivered.

Our findings highlight a targeted hiring process in which experienced nurses and outreach shelter workers were selected from existing community agencies. Comparable approaches to deploy frontline providers have been previously used. [31, 32] For example, workers of the Critical Time Intervention (CTI) strategy in New York City did not require a professional degree, only past experience caring for men who were homeless and mentally ill [31]. Likewise, in the early implementation of the Coordinated Access to Care from Hospital (CATCH) intervention, case managers supervised by their home community agencies were assigned to participating hospitals [32]. In our study, the requirement for previous experience working with vulnerable populations may have mitigated the need for a stronger onboarding process. Enhanced and consistent training, however, would have helped better prepare and empower frontline providers to embrace their roles, as they consistently described feeling like they received inadequate training and support. Insufficient training for frontline staff working in the homeless sector has been recently reported as a systemic issue in two major Canadian cities [23]. Previous literature has also shown that while efforts to upgrade staff skills in the homeless sector are historically lacking, introducing comprehensive and ongoing training for frontline team members improves worker capacity [21, 22].

A central theme in this analysis was the supportive and trusting work environment that the team self-initiated and developed. Strong relationships were created primarily through frontline members’ own ability to collaborate, not through direction from partner organizations or program management. Since most frontline members expressed frequent stress, frustration, or feelings of burn out, the need to talk openly and share expertise may have encouraged team comradery. Waegemakers Schiff & Lane [23], who reported a high rate of traumatic stress among 23 Canadian homeless sector providers, suggested that peer support had a tempering effect on traumatic symptoms among frontline workers. Similar findings have been reported for disability support workers [33] and emergency room nurses [34]. Our findings suggest that working adeptly as a team is an important coping strategy that has the potential to increase worker capacity and competency, thereby facilitating stronger program outcomes.

Individual worker attributes and organizational support also play important roles in minimizing the effects of traumatic stress on frontline staff. C2C team members shared a professional identity of commitment, resourcefulness, and pride in their work. The consistency by which these traits were identified in this study underscores the importance of intentional hiring for programs serving socially vulnerable populations. Previous research suggests that personality traits such as openness and extraversion buffer effects of workplace stress [35], and that motivation plays a role in an employee’s resilience to work demands [36]. Research has also documented the importance of training to support frontline workers and increase provider job satisfaction [23, 37]. Participants in our study emphasized the need for specific training to enhance their client practice, and asked for structured time to support emotional coping. Altogether, C2C workers were enabled in their roles through their values, attitudes about the work, and the meaning they found in clients’ achievements; however, the risky and complicated nature of working with a population that suffers disproportionate trauma calls for increased systemic and organizational support.

In our study, frontline providers felt disconnected from their managing agencies and excluded from program decision making. Research has documented that feeling isolated and unsupported by organizational leads can cause employees to feel powerless, leading to impacts on quality of care and employee turnover [21, 38–40]. Further studies are needed in the homeless service sector to explore how organizations can best support their workers. Being a grant-funded program, and a program that was new and under-developed, may have contributed to some of the experiences of disconnection. At present there is little published literature exploring the dynamic between management and frontline workers in this sector, and the influence of the funding status, however at least one study suggests that grant funding may negatively impact the commitment of organizational leadership to a program [41]. Additional research is also needed to explore organizational dynamics, levels of transparency, and engagement of frontline providers in program decisions. Our findings suggest that organizational leaders should increase connection with frontline providers by listening to and acknowledging their concerns within the program.

Participants appreciated the autonomy they were given around planning and providing client care. While frontline providers’ exclusion from program decision making presented challenges, the flexibility permitted in their practice may have lessened the effects of such perceived exclusion. Frontline providers were able to make client-centred decisions, access the expertise of their team members, and support each other’s workload.

### Limitations

While our findings are congruent with previous studies and likely relevant to various settings, they are limited to the operations of a single program that is subject to context-specific factors and systemic constraints. We conducted observations for a short time, the observer was not fully immersed into the culture, and some nuances may not be fully captured. It is also important to consider that all interviews reflect experiences and sentiments at a particular time. Though many themes were consistent across our two interview periods, the responses may have been influenced by specific dynamics at given times. Quotes from team members in management positions were intentionally excluded in the article although considered in the abstractions of themes. Finally, we did not collect data on the total length of time that participants had worked in the homeless or human service sector. Additional research is required to determine whether employment history is associated with success in health navigation and case management roles.

## Conclusion

A cohesive team of providers who value attending to vulnerable populations is a core element of C2C. Personal and professional characteristics of frontline staff and their ability to personalize care plans and coordinate long-term support for a complex population were foundational, allowing them to take on daily challenges of a demanding role. Frontline providers’ propensity for teamwork and commitment to their clients are important elements to consider when replicating similar programs in the homeless sector. Robust onboarding processes, allocating structured time for team support, and including frontline providers in decision making are also essential for optimizing client care and long-term sustainability for such programs.

## Supplementary Information

**Additional file 1. **Interview guide. Semi-structured interview guide for C2C staff.

## Data Availability

The data that support the findings of this study are not publicly available. We do not have ethical approval to make the data public or share it with other parties. Participants consented for anonymized segments of transcripts and field notes to be used in publication but did not consent for the data collected to be shared outside the research team. Hence, a de-identified dataset is not available.

## Abbreviations

C2C: Connect 2 Care
RN: Registered Nurse
HN: Health Navigator
