# Supplementary material for: Being a member of a novel transitional case management team for patients with unstable housing: an ethnographic study
Source: BMC Health Serv Res. 2022 Feb 19;22:232. doi: 10.1186/s12913-022-07590-6 (PMC8858447; doi:10.1186/s12913-022-07590-6)
Supplement: Supplementary file 1 — Additional file 1. Interview guide. Semi-structured interview guide for C2C staff. [file 12913_2022_7590_MOESM1_ESM.docx]

**Additional File 1: Interview guide**

1. Could you please tell me about your role and perceptions of the C2C program?
   1. How did you get involved with it?
   2. How long have you been working with the C2C program?
   3. How has your role within the program changed since you first joined the coordinated care team?
   4. How does your role compare to what you thought you would be doing when you were first hired as a member of the coordinated care team?
   5. How prepared did you feel for your role in the C2C?
   6. How helpful was the training you received?
      1. What additional training, if any, would be helpful?
   7. How have your views of the program changed since you first became involved with the C2C program?
2. What do you like about the C2C program?
3. What don’t you like about the C2C program?
4. What are other resources or supports you would find useful for fulfilling your role?
5. Is there any other information you’d like to share about the C2C program and your experiences working with it?
